# Incidence and locations of preoperative deep venous thrombosis (DVT) of lower extremity following tibial plateau fractures: a prospective cohort study

**DOI:** 10.1186/s13018-021-02259-y

**Published:** 2021-02-05

**Authors:** Yanbin Zhu, Wei Chen, Junyong Li, Kuo Zhao, Junzhe Zhang, Hongyu Meng, Yingze Zhang, Qi Zhang

**Affiliations:** 1 Department of Orthopaedic Surgery, The 3rd Hospital of Hebei Medical University, NO.139 Ziqiang Road, Shijiazhuang, 050051 Hebei People’s Republic of China; 2 Key Laboratory of Biomechanics of Hebei Province, Shijiazhuang, 050051 Hebei People’s Republic of China; 3 Orthopaedic Institution of Hebei Province, Shijiazhuang, 050051 Hebei People’s Republic of China; 4 Chinese Academy of Engineering, Beijing, 100088 People’s Republic of China; 5 NHC Key Laboratory of Intelligent Orthopeadic Equipment, Shijiazhuang, Hebei 050051 P.R. China

**Keywords:** Deep venous thrombosis, Tibial plateau fractures, Epidemiology, Risk factors

## Abstract

**Background:**

There is still lack of data on deep vein thrombosis (DVT) following bone trauma**.** This study aimed to determine the epidemiologic characteristics of deep venous thrombosis (DVT) of lower extremities following tibial plateau fractures.

**Methods:**

Retrospective analysis of prospectively collected data on patients presenting with tibial plateau fractures between October 2014 and December 2018 was conducted. Duplex ultrasonography (DUS) was routinely used to screen for preoperative DVT of bilateral lower extremities. Data on demographics, comorbidities, injury-related data, and laboratory biomarkers at admission were collected. Univariate analyses and multivariate logistic regression analyses were used to identify the independent risk factors associated with DVT.

**Results:**

A total of 1179 patients were included, among whom 192 (16.3%) had a preoperative DVT, with incidence rate of 1.0% for proximal and 15.3% for distal DVT. The average interval between fracture occurrence and diagnosis of DVT was 3.5 days (median, 2 days), ranging from 0 to 19 days. DVT involved the injured extremity in 166 (86.4%) patients, both the injured and uninjured extremities in 14 patients (7.3%) and only the uninjured extremity in 12 patients (6.3%). Six risk factors were identified to be associated with DVT, including gender (male vs female), hypertension, open fracture, alkaline phosphatase > 100 u/L, sodium concentration < 135 mmol/L, and D-dimer > 0.5 mg/L.

**Conclusion:**

These epidemiologic data are conducive to the individualized assessment, risk stratification, and development of targeted prevention programs.

## Background

As is well known, deep venous thrombosis (DVT) is a significant cause of morbidity, pulmonary embolism, and even mortality in almost all hospitalized patients, especially in the setting of trauma [1, 2]. Tibial plateau fracture, a commonly seen knee injury, represents 1–2% of adult fractures and 32% of peri-knee fractures [3]. The post-injury hypercoagulation state, trauma stress, and systemic inflammatory response are early factors that contributed to the occurrence of DVT. Prolonged duration of extremity elevation and limited mobility, which is needed to allow the soft tissue envelop safe for definite surgical intervention, aggravates the risk of DVT.

Extensive and deep understanding of the related risk factors is critical for prevention of occurrence of DVT, and it is of more clinical significance to distinguish between proximal and distal venous thrombosis, which allows more accurate diagnosis and management of DVT. While DVT has been extensively studied in major trauma, such as hip fracture, spinal fracture, pelvic and acetabular fracture, and multiple trauma [4–7], there is still lack of epidemiologic data on DVT following tibial plateau fracture. In most cases, the limited sample size in a single institution provided underpowered statistical analyses. Besides, the confounding covariables from multi-aspects as trauma stress, patient comorbidities, or injury itself could affect the occurrence of DVT, and their respective role has not been definitely established.

In this study, we used the prospectively collected data to evaluate the epidemiologic characteristics of preoperative DVT in tibial plateau fractures, including the incidence rate, the locations, and of DVTs and the associated risk factors.

## Methods

The methods of this study were performed in accordance with the STROCSS (Strengthening the Reporting of Cohort Studies in Surgery) guidelines. The data were extracted from the database of Surgical Site Infection in Orthopaedic Surgery (SSIOS), in which data were prospectively collected on patients undergoing orthopedic surgeries between October 1 and December 31, 2018, and surveillance and telephone follow-up were used to identify cases of surgical site infection. The SSIOS was approved by the ethics committee of the 3rd Hospital of Hebei Medical University (NO 2014-015-1) and got the informed consent of all the participants.

### Inclusion and exclusion criteria

Inclusion criteria were patients aged 18 years or older, definite diagnosis of tibial plateau fracture, and complete data available. Exclusion criteria were pathological (metastatic) or old fracture (> 3 weeks from injury), concurrent fractures in other locations, history of DVT or other thrombotic events, or current use of anticoagulants due to chronic comorbidities.

During hospitalization, all patients received basic thromboprophylaxis, consisting of chemical (low molecular weight heparin (LMWH), 2500–4100 IU once daily, subcutaneous injection) and elevation of the injured lower extremity.

### Diagnosis of DVT

DVT was diagnosed in accordance with the Guideline for the Diagnosis and Treatment of Deep Vein Thrombosis (3rd edition) proposed by the Chinese Medical Association [8]. Before the operation, routine duplex ultrasonography (DUS) scanning of the femoral common vein, superficial and deep femoral vein, popliteal vein, posterior and anterior tibial vein, and peroneal vein of bilateral lower extremities was performed to detect DVT. The positive criteria of DVT were set as non-compressibility, lumen obstruction or filling defect, lack of respiratory variation in above knee segments, and inadequate flow augmentation to calf and foot compression maneuvers [9]. DVT localized in the popliteal vein or proximally was defined as proximal DVT, and those distal to popliteal vein as distal DVT; if both distal and proximal DVT were present in one patient, he was classified in the proximal DVT group [10]. Superficial or intermuscular vein thrombosis (soleal or gastrocnemius vein thrombosis) were excluded, due to their relatively less clinical significance [11, 12].

### Data collection

The demographic data included age, gender, residence (urban or rural), body mass index (BMI), cigarette smoking, and alcohol consumption. The comorbidities included hypertension, diabetes, chronic heart disease, chronic liver disease, history of any surgery, and allergies to any medications, all of which were self-reported by patients. Injury-related data included injury mechanism (low- or high-energy trauma), open or closed fracture, fracture classification based on Schatzker classification system, and American Society of Anesthesiologists (ASA) classification. The BMI (kg/m^2^) was divided using the criteria recommended by the Chinese working group on obesity: normal (18.5–23.9), underweight (< 18.5), overweight (24.0–27.9), and obesity (≥ 28.0) [13]. Low-energy injury was defined as a fall from a standing height or a height less than 1 m, while fall from a height of 1 m or more or motor accidents as high-energy injury.

The biomarkers at admission included total protein level, albumin level, globulin, albumin/globulin, alanine transaminase, aspartate transaminase, alkaline phosphatase, lactate dehydrogenase, uric acid, fasting blood glucose level, total bilirubin, direct bilirubin, indirect bilirubin; red blood cell count, white blood cell count, neutrophil count, lymphocyte count, monocyte, hemoglobin level, hematocrit, platelet, red blood cell distribution width, platelet distribution width, mean erythrocyte volume, mean corpuscular hemoglobin, mean corpuscular hemoglobin concentration, total cholesterol level, triglyceride level, low-density lipoprotein level, high-density lipoprotein level, very low-density lipoprotein level, sodium concentration, chloride concentration, D-dimer level, and osmotic pressure.

### Statistical analysis

Continuous variables were expressed by mean and standard deviation (SD) and were evaluated by Student’s *t* test or Mann-Whitney *U* test, as appropriate. The categorical data were expressed as number and percentage (%) and were evaluated by chi-square or Fisher’s exact test, as appropriate. Multivariate logistics regression model was used to identify the independent risk factors associated with occurrence of DVT, using the stepwise backward elimination method. Variables with *p* < 0.10 were retained in the final model, and the correlation strength is indicated by odds ratio (OR) and 95% confidence interval (95% CI). The significance level was set as *p* < 0.05. Hosmer-Lemeshow (H-L) test was used to evaluate the fitting degree of the final model, and *p* > 0.05 represented the acceptable result. SPSS23.0 was used to perform all the tests (IBM, Armonk, NY, USA).

## Results

During the study period, 1179 patients were included, consisting of 742 males and 437 females, with an average of 45.6 years (Sd, 13.6; range, 18–82; median, 45.0). 64.3% (758) of the fractures were caused by high-energy trauma, and 6.0% (71) were open fractures. There were 794 (67.3%) fractures classified as types I–IV, and 385 (32.7%) as types V–VI.

One hundred ninety-two patients had a preoperative DVT, indicating an incidence rate of 16.3%. There was no thrombosis in deep femoral vein. At the other 6 veins, a total of 314 clots were found, representing an average of 1.64 (range, 1 to 4) for each patient. DVT involved the injured extremity in 166 patients, both the injured and uninjured extremities in 14 patients (7.3%) and only the uninjured extremity in 12 patients (6.3%). DVT involved proximal vein in 12 patients and distal vein in 180 patients, with incidence rate of 1.0% and 15.3%, respectively. The details were 2 in femoral common vein, 3 in superficial femoral vein, 24 in popliteal vein, 1 in anterior tibial vein, 116 in posterior tibial vein, and 168 in peroneal vein. The median time from injury to DVT was 2 days, ranging from 0 to 19 days (interquartile range (IQR), 1 to 4). 86.4% (165/192) of the DVTs were diagnosed within 7 days after the fracture, and 66.0% (126/192) were diagnosed within 2 days. Compared to those without DVTs, patients with a preoperative DVT had a significantly longer total hospitalization stay (24.8 ± 15.5 vs 19.2 ± 14.6, *p* = 0.002).

It was different between DVT and non-DVT patients in term of gender, age in continuous form, hypertension, fracture type (I–IV vs V–VI), fracture pattern (open vs close), current smoking status, ASA classification, total protein, albumin, albumin/globulin value, direct bilirubin, lactate dehydrogenase, sodium concentration, chloride concentration, fasting blood glucose, white blood cell, neutrophil count, lymphocyte count, monocyte, red blood cell count, hemoglobin, hematocrit, platelet distribution width, and D-dimer. The retails were listed in Table 1.

**Table 1:** Univariate analyses of risk factors associated with preoperative DVT following tibial plateau fracture

Variables	Number (%) of DVT (*n* = 192)	Number (%) of non-DVT (*n* = 987)	***P***
**Gender (male)**	139 (72.4)	603 (61.1)	0.003
**Age (years)**	47.4 ± 11.9	45.2 ± 13.9	0.045
18–44	81(42.2)	477 (48.3)	0.108
45–64	96 (50.0)	413 (41.8)	
65 or older	15 (7.8)	97 (9.8)	
**Living place**			0.459
Rural	126 (65.6)	603 (61.1)	
Urban	66 (34.4)	383 (38.8)	
**BMI (kg/m**^**2**^**)**	26.0 ± 2.9	25.8 ± 4.1	0.562
18.5–23.9	44 (23.0)	318 (32.2)	
< 18.5	2 (1.0)	19 (1.9)	
24.0–27.9	102 (54.0)	409 (41.4)	
≥ 28.0	44 (23.0)	242 (24.5)	
**Diabetes mellitus**	23 (12.0)	131 (13.3)	0.627
**Hypertension**	47 (24.5)	167 (16.9)	0.013
**Chronic heart disease**	14 (7.3)	42 (4.3)	0.070
**Chronic liver disease**	6 (3.1)	47 (4.8)	0.317
**History of any surgery**	19 (9.9)	116 (11.8)	0.460
**Allergy to any medications**	33 (17.2)	121 (12.3)	0.064
**Fracture type (Schatzker)**			0.022
I–IV	133 (69.3)	760 (77.0)	
V–VI	59 (30.7)	326 (23.0)	
**Mechanism (high-energy)**	120 (62.5)	638 (64.6)	0.571
**Open fracture**	22 (11.5)	49 (5.0)	0.001
**Current smoking**	37 (19.3)	129 (13.1)	0.024
**Alcohol consumption**	20 (10.4)	87 (8.8)	0.480
**Total hospital stay**	24.8 ± 15.5	19.2 ± 14.6	0.002
**ASA class**			0.000
I	29 (15.1)	138 (14.0)	
II	116 (60.4)	715 (72.4)	
III or above	47 (24.5)	134 (13.6)	
**TP (< 60 g/L)**	66 (34.4)	261 (26.4)	0.025
**ALB (< 35 g/L)**	61 (31.8)	203 (20.6)	0.001
**GLOB (< 20 g/L)**	22 (11.5)	154 (15.6)	0.140
**A/G**			0.020
1.2–2.4	164 (84.5)	883 (89.5)	
< 1.2	25 (13.0)	73 (7.4)	
> 2.4	3 (1.6)	31 (3.1)	
**ALT (> 40 U/L)**	46 (24.0)	180 (18.2)	0.065
**AST (> 35 U/L)**	33 (17.2)	134 (13.6)	0.189
**TBIL (> 21 umol/L)**	20 (10.4)	82 (8.3)	0.342
**DBIL (> 6umol/L)**	69 (35.9)	274 (27.8)	0.022
**IBIL (> 14 umol/L)**	30 (15.6)	150 (15.2)	0.880
**ALP (> 100)**	20 (10.4)	41 (4.2)	0.000
**LDH (> 250 U/L)**	64 (33.3)	214 (21.7)	0.001
**TC (> 5.2 mmol/L)**	17 (8.9)	121 (12.3)	0.179
**TG (> 1.7 mmol/L)**	33 (17.2)	167 (16.9)	0.928
**HDL-C (< 1.1 mmol/L)**	96 (50.0)	388(39.3)	0.006
**LDL-C (> 3.37 mmol/L)**	27 (14.1)	135(13.7)	0.887
**VLDL (> 0.78 mmol/L)**	32 (16.7)	162 (16.4)	0.931
**Na+ (< 135 mmol/L)**	80 (41.7)	286 (29.0)	0.001
**CL− (mmol/L)**			0.007
99–110	149 (77.6)	847 (85.8)	
< 99	40 (20.8)	121 (12.3)	
> 110	3 (1.6)	19 (1.9)	
**GLU (> 6.1 mmol/L)**	82 (42.7)	334 (33.8)	0.019
**UA (> upper limit)**	8 (4.2)	78 (7.9)	0.069
WBC (> 10*10^**9**^**/L)**	81 (42.2)	310 (31.4)	0.004
NEUT (> 6.3*10^**9**^**/L)**	116 (60.4)	468 (47.4)	0.003
LYM (< 1.1*10^**9**^**/L)**	57 (29.7)	283 (28.7)	0.776
MON (> 0.6*10^**9**^**/L)**	130 (67.7)	592 (60.0)	0.044
**RBC < lower limit**	103 (53.6)	388 (39.3)	0.000
**HGB < lower limit**	131 (68.2)	526 (53.3)	0.000
**HCT < lower limit**	145 (75.5)	605 (61.3)	0.000
**MCV (fL)**			0.737
82–100	176 (91.7)	918 (93.0)	
< 82	9 (4.7)	35 (3.5)	
> 100	7 (3.6)	34 (3.4)	
**MCH (pg)**			0.658
27–34	176 (91.7)	903 (91.5)	
< 27	10 (5.2)	42 (4.3)	
> 34	6 (3.1)	42 (4.3)	
**MCHC (< 316 g/L)**			0.759
316–354	179 (93.2)	919 (93.1)	
< 316	7 (3.6)	29 (2.9)	
> 354	6 (3.1)	39 (4.0)	
**PDW (%)**			0.007
12–18.1	154 (80.2)	871 (88.2)	
< 12	34 (17.7)	98 (9.9)	
> 18.1	4 (2.1)	18 (1.8)	
**D-dimer** (> 0.5 mg/L**)**	39 (20.3)	153 (79.7)	0.000
**Anion gap (**mmol/L**)**			0.608
8–16	147 (76.6)	773 (78.3)	
< 8	43 (22.4)	197 (20.0)	
> 16	2 (1.0)	17 (1.7)	
**OSM < 260 mOsm/L**	22 (11.5)	95 (9.6)	0.437

*BMI* body mass index, *ASA* American Society of Anesthesiologists, *TP* total protein, *ALB* albumin, *A/G*, albumin/globulin, *ALT* alanine transaminase, *AST* aspartate transaminase, *TBIL* total bilirubin, *DBIL* direct bilirubin, *IBIL* indirect bilirubin, *ALP* alkaline phosphatase, *LDH* lactate dehydrogenase, *TC* total cholesterol, *TG* triglycerides, *HDL-C* high-density lipoprotein cholesterol, *LDL-C* low-density lipoprotein cholesterol, *VLDC* very low-density lipoprotein, *GLU* urine glucose, *UA* uric acid, *WBC* white blood cell, *NEU* neutrophil count, *LYM* lymphocyte, *MON* monocyte count, *RBC* red blood cell; reference range: female, 3.5–5.0 × 10^12^/L; males, 4.0–5.5 × 10^12^/L; *HGB* hemoglobin; reference range: females, 110–150 g/L; males, 120–160 g/L, *HCT* hematocrit, *MCV* mean corpuscular volume, *MCH* mean corpuscular hemoglobin, *MCHC* mean corpuscular hemoglobin concentration, *PDW* platelet distribution width, *D-dimer*, *OSM* osmotic pressure

Entered in the multivariate model were the above 24 variables, together with history of heart disease (*p* = 0.070), allergy to any medication (*p* = 0.064), alanine transaminase (*p* = 0.065), and uric acid (*p* = 0.069). Six risk factors were identified to be associated with DVT, including gender (male vs female), hypertension, open fracture, alkaline phosphatase > 100 u/L, sodium concentration < 135 mmol/L, and D-dimer > 0.5 mg/L (Table 2). The H-L test demonstrated the good fitness of the final model (*X*^2^ = 3.343, *p* = 0.911; Nagelkerke *R*^2^ = 0.108).

**Table 2:** Multivariate analysis of factors associated with DVT following tibial plateau fracture

Variables	OR	95% CI (lower limit)	95% CI (upper limit)	***P***
Male (vs female)	1.71	1.20	2.44	0.003
Hypertension	1.49	1.10	2.01	0.009
Open fracture (vs close)	2.03	1.16	3.56	0.014
ALP (> 100 u/L)	2.20	1.21	3.99	0.010
Na+ (< 135 mmol/L)	1.42	1.01	1.98	0.043
D-dimer (> 0.5 mg/L)	2.73	1.87	4.00	< 0.001

## Discussion

There is still lack of data on epidemiologic characteristics of DVTs following tibial plateau fractures. In this study, we used the prospectively collected data to address this issue. We found the overall incidence of DVT following tibial plateau fracture was 16.3%, with 1.0% for proximal and 15.3% for distal DVT; 86.4% of DVTs were diagnosed within 7 days after the injury and 66.0% within 2 days. Six risk factors were identified to be associated with DVT, including males (vs female), hypertension, open fracture, alkaline phosphatase > 100 u/L, sodium concentration < 135 mmol/L, and D-dimer > 0.5 mg/L. There have been some reports on the DVT in lower extremity fractures, with greatly variable rates, ranging from 2.6 to 86.0% [10, 14–16], and study design, heterogeneity of included participants, and the presence or not of prophylaxis account for this big difference. Specified at tibial plateau fracture, there were three studies that reported the incidence of preoperative DVT. Wang et al. [15] found the incidence rate of preoperative DVT was 17.3% in 336 patients with tibial plateau fractures. Li et al. [17] reported the rate of 29.1% in 55 patients with high-energy AO-C type of tibial plateau fractures. In a study of 1825 patients with isolated lower extremity fractures, the authors reported the rate of preoperative DVT following tibial plateau fractures was 23.9%. In contrast, we found a slightly lower incidence rate (16.3%), and this may be due to the fact that our cohort included patients at a relatively younger age (45.6 vs 62.8 years in Wang et al. [10]), and included fracture caused both by low- and high-energy injury (vs only high-energy in Li et al.’s [17]). Another reason could be that intermuscular vein thrombosis were excluded in this study due to their less clinical significance, whereas the study by Wang et al. [15] included DVTs in the intermuscular vein and another study did not specifically mentioned that [10].

In this study, DVT involved injured extremity in 86.4%, bilateral extremity in 7.3%, and only the uninjured extremity in 6.3% of patients who had developed DVT. This finding was consistent with that by Wang et al. [10], who reported the corresponding figures of 83.6%, 5.4%, and 11.0% in patients who sustained lower extremity fractures. It was likely that the vascular endothelial injury due to bone trauma, systemic hypercoagulability, and the immobilization after fracture combined to result in the predominant proportion of DVT in injured extremity. We also found that proximal DVT took a low proportion (6.3%, 12/192) and the incidence was 1.0% (12/1179), much lower than that following hip fracture, femoral shaft fracture [18, 19]. This difference might be explained by the correlation of different fracture sites and locations of DVT, with a possibility of higher risk of proximal DVT in fractures more proximal to hip joint. Despite this, we should not ignore the distal DVTs, because they demonstrated to present a similar risk for pulmonary embolism as proximal DVT [20].

The positive relationship between delayed surgical treatment and higher risk of preoperative DVT following bone trauma has been extensively studied [10, 21]. In this study, DVT was diagnosed at a mean of 3.5 days (median, 2 days) after injury, and 86.4% were diagnosed within 7 days. This can be explained by the finding by Wilson et al. [22] that the blood coagulation was highest in 3–7 days after fracture. In this study, the mean interval between injury and operation was 7.4 days, and only 24.3% of patients were operated within 3 days, and 11.5% after 7 days. That means 64.2% of patients are right in peak blood coagulation status; and for those patients, therefore, initiation of anticoagulation prophylaxis as soon as possible is necessary.

D-dimer in plasma reflects the secondary increased fibrinolytic activity and the hypercoagulability, which is a well-established sensitive marker of thrombotic events, although with a poor specificity [23]. This study reconfirmed that elevated D-dimer level (> 0.5 mg/L) was associated with 2.73-fold increased risk of DVT. In some studies, authors observed the higher incidence rate of DVT in males than in females [21, 24], but in others, the incidence was not different in gender [25]. In this study, males had a higher incidence of DVT and demonstrated to be an independent factor, but the mechanism remains unclear.

Hypertension as an independent risk factor for DVT was well discussed in literature, including a recent meta-analysis [26], which was reconfirmed in the present study. Generally, the high-energy trauma induced the response or higher level of stress response, or inflammatory/immune response, theoretically, was associated with increased risk of DVT. However, in this study, high- and low-energy trauma did not differ in incidence of DVT. It was possible that this effect was counteracted by the age, which was older in patients with low-energy trauma. Wang et al. [15] suggested the fall from a height, rather than fracture type or multiple trauma, is an independent factor for DVT. In this study, open injury, rather than Schatzker V–VI type or high-energy trauma was identified as a significant factor for DVT. Therefore, we could infer that soft tissue injury and the blood flow state play a key role in the formation of DVT.

Hyponatremia is common among patients sustaining trauma, taking a proportion of 31.0% in this study. Sodium imbalances, either hyper- or hyponatremia, was associated with the increased risk of venous thromboembolism or mortality [27]. In this study, due to the limited sample of patient with hypernatremia (26 patients, no DVT in any patient), we only identified the independent association of hyponatremia with DVT (OR, 1.42). It is well known that elevated plasma ALP level reflected the increased activity of osteoblasts, but its role in development of DVT is not illuminated. The researchers found ALP level was positively related to injury severity score (ISS) [28] in bone fractures and to fracture severity [29], which could partly explain the higher incidence of DVT in patients with elevated ALP level.

There were several limitations in this study. Firstly, as other multivariate analyses, we could not include all the potential factors that affect the occurrence of DVT, such as duration of immobilization of the injured extremity [11]. Secondly, for some uncommon medical conditions or comorbidities such as chronic nephrosis or long-term use of glucocorticoid, it is almost impossible to confirm their association with DVT. Third, we identified the association rather than the causation between variables and DVT; therefore, these results should be interpreted with caution.

## Conclusions

In summary, the incidence of preoperative DVT following tibial plateau fracture was 16.3%, and 86.4% of DVTs were diagnosed within 7 days after the injury and 66.0% within 2 days. Males, hypertension, open fracture, alkaline phosphatase > 100 u/L, sodium concentration < 135 mmol/L, and D-dimer > 0.5 mg/L were identified to be associated with DVT. These epidemiologic data are conducive to the individualized assessment, risk stratification, and development of targeted prevention programs.

## Data Availability

All the data will be available upon motivated request to the corresponding author of the present paper.

## Abbreviations

DVT: Deep venous thrombosis
STROCSS: Strengthening the Reporting of Cohort Studies in Surgery
SSIOS: Surgical Site Infection in Orthopaedic Surgery
LMWH: Low molecular weight heparin
DUS: Duplex ultrasonography
STROCSS: Strengthening the Reporting of Cohort Studies in Surgery
BMI: Body mass index
OR: Odds ratio
CI: Confidence interval
SD: Standard deviation
